# Preclinical Evaluation of Nitroxide-Functionalised Ciprofloxacin as a Novel Antibiofilm Drug Hybrid for Urinary Tract Infections

**DOI:** 10.3390/antibiotics12101479

**Published:** 2023-09-22

**Authors:** Sophia Hawas, Jilong Qin, Sandra Wiedbrauk, Kathryn Fairfull-Smith, Makrina Totsika

**Affiliations:** 1 Centre for Immunology and Infection Control, School of Biomedical Sciences, Faculty of Health, Queensland University of Technology, Brisbane, QLD 4006, Australia; sophia.hawas@hdr.qut.edu.au (S.H.); jilong.qin@qut.edu.au (J.Q.); 2 Max Planck Queensland Centre, Queensland University of Technology, Brisbane, QLD 4059, Australia; 3 School of Chemistry and Physics, Faculty of Science, Queensland University of Technology, Brisbane, QLD 4000, Australia; sandra.wiedbrauk@qut.edu.au (S.W.); k.fairfull-smith@qut.edu.au (K.F.-S.); 4 Centre for Materials Science, Queensland University of Technology, Brisbane, QLD 4000, Australia

**Keywords:** bladder, urinary tract infection, UPEC, bacterial biofilms, catheters, nitroxides, antibiotic resistance, fluoroquinolones, therapeutics

## Abstract

Urinary tract infections (UTIs) are the second most common bacterial infection with high recurrence rates and can involve biofilm formation on patient catheters. Biofilms are inherently tolerant to antimicrobials, making them difficult to eradicate. Many antibiofilm agents alone do not have bactericidal activity; therefore, linking them to antibiotics is a promising antibiofilm strategy. However, many of these hybrid agents have not been tested in relevant preclinical settings, limiting their potential for clinical translation. Here, we evaluate a ciprofloxacin di-nitroxide hybrid (CDN11), previously reported to have antibiofilm activity against uropathogenic *Escherichia coli* (UPEC) strain UTI89 in vitro, as a potential UTI therapeutic using multiple preclinical models that reflect various aspects of UTI pathogenesis. We report improved in vitro activity over the parent drug ciprofloxacin against mature UTI89 biofilms formed inside polyethylene catheters. In bladder cell monolayers infected with UTI89, treatment with CDN11 afforded significant reduction in bacterial titers, including intracellular UPEC. Infected mouse bladders containing biofilm-like intracellular reservoirs of UPEC UTI89 showed decreased bacterial loads after ex vivo bladder treatment with CDN11. Activity for CDN11 was reported across different models of UTI, showcasing nitroxide–antibiotic hybridization as a promising antibiofilm approach. The pipeline we described here could be readily used in testing other new therapeutic compounds, fast-tracking the development of novel antibiofilm therapeutics.

## 1. Introduction

Urinary tract infections (UTIs) are one of the most common bacterial infections with a high rate of recurrence (~30%). UTI clinical syndromes include cystitis, pyelonephritis, urosepsis and asymptomatic bacteriuria (ASB) [1]. Hospital-acquired UTIs represent high-burden nosocomial infections, especially in patients undergoing long periods of urinary catheterisation, known as catheter-associated UTI (CAUTI) [2]. Uropathogenic *Escherichia coli* (UPEC) are the major causative agents of UTIs, capable of persisting intracellularly and forming biofilms [3]. Biofilms are sessile multicellular communities of bacteria embedded in a self-produced extracellular matrix, capable of forming on both biotic and abiotic surfaces. In UTI, UPEC also form biofilm-like intracellular bacterial communities (IBCs) inside bladder cells during early stages of the infection [4], or in the case of CAUTI, form biofilms inside the lumen of urinary catheters [5,6]. Biofilms are inherently tolerant to environmental stress, antimicrobials, and host immune responses, making them difficult to eradicate [7,8,9,10]. Following IBC formation during acute UTI, UPEC can persist in urinary tissues for longer periods in the form of intracellular quiescent reservoirs (QIRs) or in the case of CAUTI, as residual (persister) bacterial cells remaining attached on the catheter surface after antibiotic therapy; both forms share characteristics with biofilms and are intrinsically difficult to eradicate [11,12,13].

As such, antibiotic treatment alone for biofilm infections is becoming increasingly unsuccessful, resulting in treatment failure or recurrence. Completely eradicating biofilms with antibiotics alone is extremely challenging, let alone achieving clinically significant bacterial reduction. The precise mechanism by which antibiotics fall short in fully eradicating bacterial biofilms remains an open question [7,14,15]. Restricted drug penetration resulting from protective extracellular matrices, varying metabolic states, and the presence of persister cells within biofilms are all proposed to contribute to the high antibiotic tolerance of biofilms [16]. 

Currently, molecules known as biofilm dispersal agents are being tested as antibiofilm therapeutics [17]. Nitroxides, stable nitric oxide (NO) mimics, are included in this category, having been reported to reduce the biofilm biomass of various Gram-negative species in vitro [18]. As nitroxides are stable (unlike NO) and thus more amenable to chemical manipulation, they have been successfully used in the synthesis of functionalised antibiotics and biocides [19,20,21,22], ensuring co-delivery with antimicrobials. Some nitroxide-antibiotic hybrids were reported to have improved biofilm eradication activity compared to the parent drug, with activity reported against several clinically important Gram-negative and Gram-positive bacterial biofilms, displaying their potential as antibiofilm drugs [19,22]. Biofilm eradication by these hybrid drugs is likely achieved by simultaneous bacterial dispersal and killing (induced by the nitroxide and antibiotic/biocide moieties, respectively). This makes them ideal candidates for treating clinical biofilms [23], as using biofilm dispersal agents alone (or even in combination with antibiotics, but unlinked) may pose a risk for the seeding of new infection sites through viable dispersed cells [24]. However, a major gap in the clinical development of nitroxide hybrid drugs is the lack of preclinical testing in disease-relevant models involving not only bacterial biofilms but also host and environmental factors such as immune responses, cell/tissue penetration, and biofilms formed on disease-related surfaces, such as catheters. 

**Figure 1:**
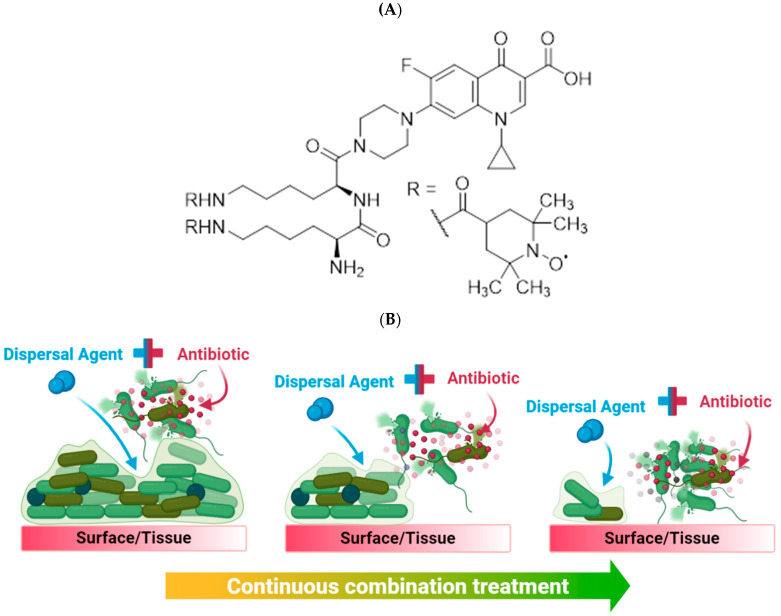
(**A**) Chemical structure of ciprofloxacin–dinitroxide hybrid 11 (CDN11), and (**B**) theorised mechanism of action for hybrid drugs containing a biofilm dispersal agent and an antibiotic, adapted from [28]. We hypothesise that the nitroxide moiety disrupts the biofilm structure, allowing the antibiotic moiety to access bacterial cells and kill them. Continuous hybrid treatment slowly reduces biofilm biomass until complete eradication.

Here, we evaluate CDN11, a ciprofloxacin–dinitroxide hybrid previously reported to have antibiofilm activity against *E. coli* in vitro [22] (Figure 1), as a potential UTI therapeutic in relevant preclinical models. Ciprofloxacin is a commonly prescribed fluoroquinolone antibiotic for both uncomplicated and complicated UTIs [25], with diminishing efficacy over the past decade due to the rise of fluoroquinolone resistant UPEC lineages [26,27]. Many antibiofilm agents undergoing preclinical evaluation fail to replicate important biofilm infection niches, such as formation on clinically relevant surfaces (i.e., catheters), or fail to account for the presence of host factors, such as tissue structure and immune responses. Here we address those shortcomings by evaluating CDN11 in a suite of relevant preclinical models for UTI and biofilm infection.

## 2. Results

### 2.1. CDN11 Eradicates UPEC Biofilms Formed on Urinary Catheters In Vitro

**Figure 2:**
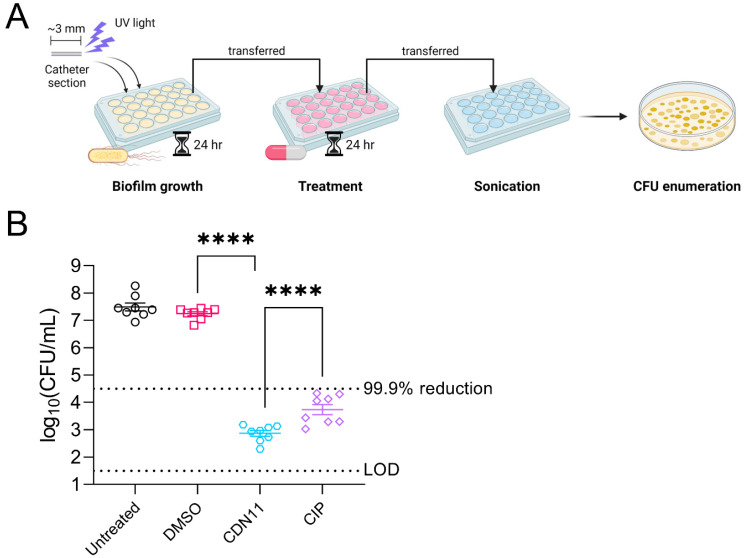
**CDN11 has potent antibiofilm activity against UPEC biofilms formed inside urinary catheters in vitro**. (**A**) Schematic of the in vitro catheter biofilm model. The ~3 mm sections of UV-sterilised (lightning symbol) polyethylene catheter were incubated with 4 × 10^6^ CFU of UTI89 in LB media for 24 h followed by 24 h of treatment with 400 μM CDN11, 400 μM ciprofloxacin, 3.5% DMSO (vehicle), or media only (MH, Untreated), before disruption with mild sonication for colony forming unit (CFU) enumeration. (**B**) Biofilm recovered bacteria from each treatment group were plotted as log10(CFU/mL) from eight biological replicates, with group means and standard deviation shown by horizontal lines and error bars, respectively. Differences between group means were calculated using ordinary one-way ANOVA with Dunn’s correction, and significance was shown as **** *p* ≤ 0.0001. The technical limit of CFU detection (LOD) and the 99.9% CFU reduction cut-off (compared to vehicle control) are marked by dotted lines.

Dinitroxide–ciprofloxacin hybrid CDN11 was synthesised as previously described [22]. The minimum inhibitory concentration (MIC) and minimum biofilm eradication concentration (MBEC) of CDN11 against reference cystitis UPEC isolate UTI89 was confirmed as ≤10.5 μM and ≤400 μM, respectively, in agreement with previous reports [22] (Supplementary Figure S1). To simulate UPEC biofilm formation in the context of CAUTI, mature biofilms of UTI89 were established at high cell density inside polyethylene catheters and then treated with antibiotics. After 24 h of treatment with CDN11 at 400 μM (MBEC concentration), viable CFU remaining in the biofilm were reduced by >4 logs compared to vehicle treated (DMSO) and untreated biofilm controls (Figure 2). Thus, CDN11 antibiofilm activity in the catheter biofilm treatment model exceeded the 99.9% reduction in CFU cut-off used to define biofilm eradication in vitro and was significantly better than ciprofloxacin administered at the same concentration (*p* ≤ 0.0001, Figure 2). 

### 2.2. In Vitro Treatment of Infected Human Bladder Cells with CDN11 Reduces Extracellular and Intracellular UPEC Titers

**Figure 3:**
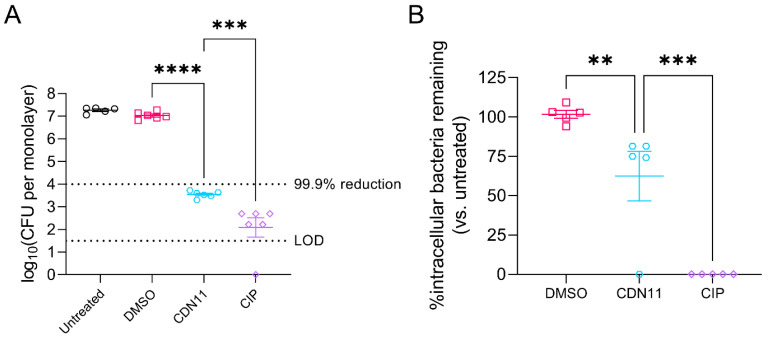
**CDN11 controls extracellular and intracellular UPEC during in vitro human bladder cell infection**. Confluent monolayers of T24 bladder cells were incubated with UPEC strain UTI89 at a multiplicity of infection (MOI) of 10 for 1 h followed by (**A**) 2 h of treatment with either 400 μM CDN11, 400 μM ciprofloxacin, an equivalent percentage of DMSO instead of CDN11 (vehicle), or media only (RPMI (Roswell Park Memorial Institute) + 5% FBS (foetal bovine serum), Untreated) or (**B**) 1 h of treatment with 200 μg/mL gentamicin to first eliminate extracellular bacteria, followed by 2 h of treatment with either 400 μM CDN11, 400 μM ciprofloxacin, an equivalent percentage of DMSO instead of CDN11 (vehicle), or media only (RPMI + 5% FBS). Bacteria recovered from infected monolayers were plotted per treatment group as (**A**) log10(CFU/mL) or (**B**) % intracellular bacteria compared to the media only control group. Data shown are from five to six biological repeats, with means and standard deviation shown using horizontal lines and error bars, respectively. Differences between group means were calculated using ordinary one-way ANOVA with Dunn’s correction, and significance was shown as ** *p* ≤ 0.01, *** *p* ≤ 0.001, and **** *p* ≤ 0.0001. The technical limit of CFU detection (LOD) and the 99.9% CFU reduction cut-off (compared to vehicle control) are marked with dotted lines.

We next tested whether CDN11 could effectively control UPEC infection of human cells in vitro. Human bladder epithelial cell (T24) monolayers infected with UPEC strain UTI89 for 1 h at a multiplicity of infection (MOI) of 10 were treated for 2 h with antibiotics. CDN11 at 400 μM significantly reduced the total CFU recovered from infected monolayers compared to untreated and vehicle treated controls (mean reduction of ≥3.5 logs, Figure 3A). Similarly, ciprofloxacin afforded a significant reduction in the total UPEC titer and cleared infection in one of eight treated monolayers (Figure 3A). 

UPEC infection of T24 human bladder cells involves both attachment (extracellular bacteria) and invasion (intracellular bacteria) of bladder cells, resembling UPEC pathogenesis during bladder infection (cystitis) of mice and humans [29]. To specifically test CDN11 activity against intracellular UPEC reservoirs, we performed gentamicin protection assays [30], whereby the infected T24 cell monolayers were first treated with gentamicin to selectively eliminate extracellular UPEC followed by test antibiotics. Despite its large molecular size (>900 Da), CDN11 displayed activity against intracellular UPEC when administered at 400 μM, reducing the mean intracellular titer by ~40% compared to untreated and vehicle controls and clearing the infection in one of five treated monolayers (Figure 3B). Ciprofloxacin-treated monolayers were completely cleared of intracellular UPEC, with no detectable colony forming units (CFU) recovered from all monolayers examined (Figure 3B).

### 2.3. Ex Vivo Treatment of Infected Mouse Bladders with CDN11 Reduces Intracellular UPEC Reservoirs

**Figure 4:**
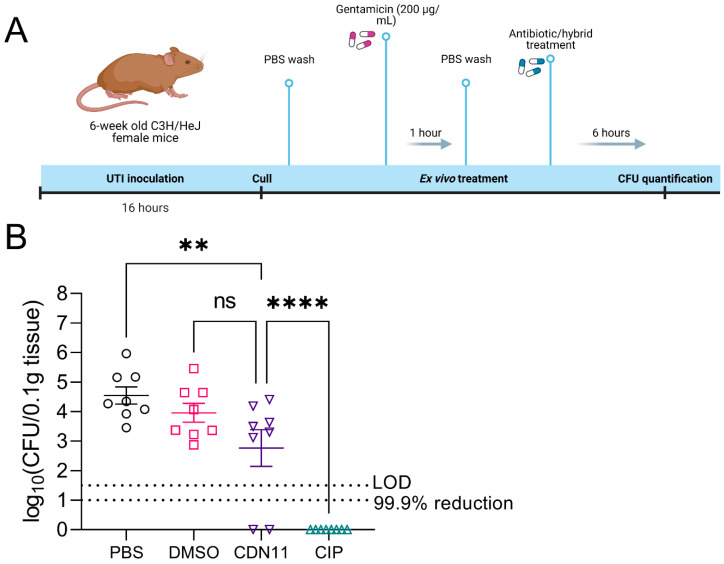
**CDN11 reduces intracellular UPEC reservoirs in infected mouse bladders after 6 h of treatment ex vivo.** (**A**) Schematic overview of acute UTI mouse model ex vivo bladder treatment timeline. Six-week-old female C3H/HeJ mice were inoculated with 1 × 10^8^ CFU of UPEC strain UTI89 and allowed to establish acute UTI for 16 h. Mice were then culled, bladders extracted and bisected, followed by 1 h of treatment with gentamicin, and then 6 h of treatment with either 400 μM CDN11, ciprofloxacin, an equivalent percentage of DMSO instead of CDN11 (vehicle), or phosphate-buffered saline (PBS) only. Bladder halves were homogenised post treatment, serially diluted, and CFU enumerated. (**B**) Bacteria recovered from mouse bladders were plotted per treatment group as log_10_(CFU/0.1 g tissue). Data shown are from eight mice per group with means and standard deviation shown by horizontal lines and error bars, respectively. Differences between group means were calculated using ordinary one-way ANOVA with Dunn’s correction, and significance was shown as **ns** not significant, ** *p* ≤ 0.01, **** *p* ≤ 0.0001. The technical limit of CFU detection (LOD) and the 99.9% CFU reduction cut-off (compared to vehicle control) are marked by dotted lines.

To evaluate early preclinical efficacy for CDN11 as a UTI therapeutic, we next tested its activity against intracellular UPEC reservoirs that are established in the bladder of mice during experimental acute UTI. Infected bladders collected from female C3H/HeJ mice with acute cystitis established by UPEC strain UTI89 were treated ex vivo with gentamicin to eliminate extracellular (luminal) UPEC, followed by treatment with test antibiotics (Figure 4A). CDN11 treatment at 400 μM completely cleared UPEC from 25% of the infected bladder tissues, and significantly reduced the mean intracellular CFU of the group compared to untreated controls (Figure 4B). In comparison, ciprofloxacin treatment cleared intracellular UPEC from all bladder tissues examined (Figure 4B). 

### 2.4. CDN11 Displays Activity against Biofilms of Fluoroquinolone-Sensitive but Not Fluoroquinolone Resistant UPEC

**Figure 5:**
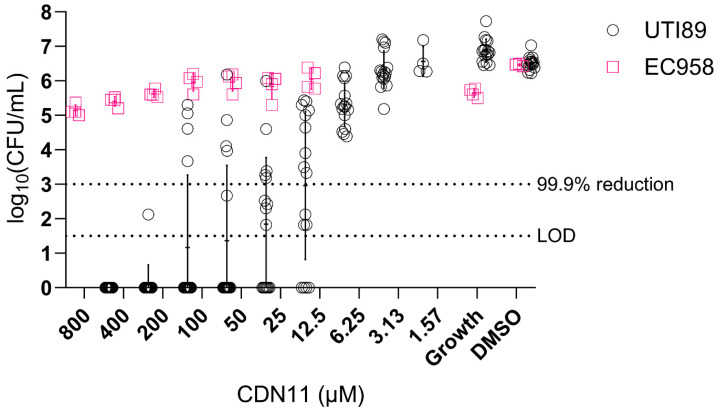
**EC958 planktonic and biofilm cells are resistant to CDN11**. Minimum biofilm eradication concentration (MBEC) values are reported as complete biofilm eradication (concentration at which there is no recoverable viable biofilm CFU). UTI89 and EC958 biofilms were grown for 24 h using the Calgary Biofilm Device at 37 °C with shaking, followed by treatment for 24 h in CDN11 at 37 °C with shaking. Biofilms were disrupted with mild sonication for CFU enumeration. Enumerated bacterial CFU was plotted as log10(CFU/mL). Data represent n = 16 (UTI89) and n = 4 (EC958) biological repeats with means and standard deviation shown. The technical limit of CFU detection (LOD) and the 99.9% CFU reduction cut-off (compared to vehicle control) are marked using dotted lines.

UTIs are increasingly caused by multidrug resistant UPEC lineages [26]. In particular, *E. coli* sequence type 131 (ST131) is the most recent globally disseminated lineage of multidrug resistant (MDR) UPEC causing UTIs and bloodstream infections, with most ST131 strains being resistant to fluoroquinolones and beta-lactam antibiotics. To investigate whether CDN11 retains antibiofilm activity against MDR UPEC, we compared its activity against UTI89 (ciprofloxacin sensitive) and EC958 biofilms, the latter being a representative ST131 MDR UPEC strain (ciprofloxacin resistant) [31]. While CDN11 displayed a biofilm CFU reduction against UTI89 in a dose-dependent manner and at concentrations as low as 12.5 μM, it did not impact established EC958 biofilms even at the highest concentration tested (Figure 5). As EC958 harbours target-modifying mutations and plasmid-encoded enzymes that grant it resistance to fluoroquinolones, this finding suggests that CDN11 shares target specificity in both planktonic and in biofilm cells with its parental antibiotic ciprofloxacin [32].

## 3. Discussion

This study evaluated a new hybrid antibiotic as a UTI therapeutic in various preclinical models that replicate clinically important aspects of the disease. Currently, the only therapeutics that treat the causative agents of UTIs are antibiotics. Biofilms formed on patient catheters However, are difficult to treat with antibiotics alone, and are often hard to eradicate due to high antibiotic tolerance [33]. During UTIs, the formation of biofilms and intracellular bacterial reservoirs contribute to disease persistence and recurrence. Greater emphasis should be placed on targeting biofilms for treating high-burden infections like UTIs. CDN11 is an antibiotic conjugate designed to target biofilm bacteria through its nitroxide moieties that were previously demonstrated to display biofilm dispersal activity [18,34]. Here, we report that the administration of CDN11 can effectively control UPEC infection in relevant preclinical models, significantly reducing the overall bacterial burden associated with infection on both biotic and abiotic surfaces, and in some cases, even clearing UPEC from infected cells and tissues. Our study is the first to showcase the potential of this hybrid antibiotic as a new UTI therapeutic.

The Calgary biofilm device (biofilms cultured on pegs) is widely used as a simple high-throughput assay to assess the antibiofilm activity of new potential therapeutics [35]. Using this assay, we determined the MBEC value of CDN11 as the minimum concentration required to fully eradicate UPEC biofilms (i.e., kill 100% of cells) not reduce 99.9% of the cells compared to growth controls, which is defined as the biofilm bactericidal concentration (BBC) and is often used interchangeably with MBEC [36]. This concentration guided our downstream testing in the biofilm models of UPEC in the context of UTI. We were able to translate our findings from the peg model to the catheter model, which more closely replicates aspects of UTI disease, such as biofilm formation on patient catheters in CAUTI. Using concentrations and treatment durations derived from the peg model, we noted similar trends between in vitro models. While catheter biofilms were not completely eradicated by CDN11 treatment at 1 × MBEC, the observed reduction in biofilm CFU was significant (over 4-logs), bringing the remaining CFU very close to the limit of detection for this assay, which is characteristically low (~35 CFU/peg). The in vitro catheter model simulates some clinically relevant factors of CAUTI, such as replicating bacterial titers seen on patient catheters and physicochemical interactions with catheter materials. The latter makes it an especially useful model for testing different catheter coatings that may contain antimicrobial compounds to limit biofilm formation or disrupt established biofilms [37]. However, the catheter model has limitations in representing important host factors during CAUTI, such as fibrinogen deposition and the recruitment of inflammatory immune cells [13,38].

In order to replicate more host–pathogen interactions during in vitro testing, we included a human cell infection model. UPEC in vitro infection of human bladder cells is commonly employed in UPEC pathogenesis studies and has been shown to recapitulate the key initial steps in UPEC pathogenesis in the urinary tract: initial FimH-mediated adherence to epithelial cells followed by UPEC uptake into the cell and intracellular persistence [30,39,40]. Important host factors necessary for this initial adhesion step are the mannosylated uroplakins present on the surface of bladder epithelial cells [41] and proinflammatory cytokines IL-6 and IL-8, which are responsible for the recruitment of immune cells to the bladder [42,43], both of which can be recapitulated using the T24 bladder cell line [44,45]. During UTIs, UPEC strains such as UTI89 are able to persist inside host cells, so by using the gentamicin protection assay model [30], we can test for compound activity specifically against intracellular bacteria. In these models, UPEC are mostly planktonic, but have been observed to form IBC-like structures [46]. While these have largely been observed in mouse UTI models and patient urine [4,47], several in vitro culture models have reported the presence of structures similar to IBCs [30,48]. CDN11 showed antimicrobial activity against both planktonic and biofilm-like UPEC in the human cell infection model, suggesting that it can penetrate mammalian cells to access intracellular bacteria, albeit less effectively than ciprofloxacin. 

Cell culture models can also discern potential compound cytotoxicity, an important step before progressing to ex vivo and in vivo testing. In a previous study, CDN11 exhibited no cytotoxicity to T24 bladder cells even up to a concentration of 500 μM [22], which exceeds the concentration used here to treat infected T24 cells. We did, however, note some morphological changes to T24 monolayers (cell rounding) that appears to be due partly to the vehicle (DMSO) used, which has been previously observed in other studies using bladder and other cell lines [49,50]. While commonly used cytotoxicity assays, like the lactate dehydrogenase (LDH) assay, can inform about a compound’s mammalian cell toxicity, they are not indicative of alterations to cell–cell interactions. These are important in the context of many mucosal infections, including UTI, and have important implications: for example, in maintaining bladder tissue integrity during infection, which is important for limiting extensive UPEC invasion during UTIs [51,52]. Investigating drug effects on cell–cell interactions in this model could screen out compounds that may disrupt the bladder architecture before they progress to in vivo testing.

In vivo infection models provide a powerful preclinical model for testing UTI therapeutics that replicate interactions between multiple tissues/cell types, UPEC (extracellular and intracellular reservoirs), the candidate compound, and host physiology. The mouse UTI model is widely used in the field to investigate host–pathogen interactions and potential drug candidates [53]. Here we treated infected mouse bladders for 6 h with CDN11 to simulate the half-life of ciprofloxacin in the human body, while the dosage (1 × MBEC; 400 μM) roughly equated to half the therapeutic ciprofloxacin dose commonly prescribed to adults for acute UTI [54,55]. The mouse UTI model can be adapted to study both acute and chronic UTI and allows for many measurable outcomes, such as bacterial load in urine and urinary organs, immune responses, and tissue morphology changes. In the mouse UTI model, UPEC can be found as free-living (planktonic) extracellular bacteria in an IBC (biofilm) or as filamentous cells upon exiting infected urothelial cells [4,56]. Using a model that incorporated filamentous UPEC, QIRs, recruited immune cells (which release nitric oxide), and the bladder’s tight cell layer architecture would provide insights into how the compound might interact with host responses and penetrate tissue layers, which would provide clinicians and researchers alike with more detailed information about a candidate compound’s activity [11,56,57]. We noted that throughout all our testing of CDN11, it retained antibiofilm activity in in vitro and ex vivo treatment models.

We observed that CDN11 is a potent antibiofilm agent in in vitro models, outperforming ciprofloxacin in both peg and catheter models, despite its lowered antibiotic activity against planktonic UTI89. We further probed CDN11’s antibiofilm activity by testing its activity against the ciprofloxacin-resistant strain EC958. Both planktonic and biofilm EC958 were resistant to high concentrations of CDN11 (>800 μM for MIC and MBEC), suggesting that CDN11’s bactericidal mode of action is the same against planktonic and biofilm bacteria. Loss of activity against ciprofloxacin resistant strains also suggests that CDN11 still targets the same enzymes as ciprofloxacin. Given that nitroxides alone are not bactericidal [34], it is most likely the dual action of the nitroxide and antibiotic that is responsible for the enhanced activity seen against UTI89 biofilms, which are ciprofloxacin-sensitive. Thus, CDN11 is a potent antibiofilm agent against ciprofloxacin-sensitive bacterial biofilms outside of the host, outperforming antibiotics alone.

We observed less CDN11 activity in models where biofilms formed on biotic surfaces, and in particular intracellularly (human cell infection assays and infected mouse bladder ex vivo treatment). Comparing CDN11 to ciprofloxacin, CDN11 has a much higher molecular weight (952.20 g mol^−1^ vs. 331.34 g mol^−1^), suggesting that it may have reduced the cell permeability of both bacterial and mammalian cells [58,59]. Other factors known to reduce cell permeability and tissue penetration include a higher polar surface area and bond flexibility [58], both of which are increased for CDN11 compared to ciprofloxacin. Ciprofloxacin’s bactericidal activity results from its binding and the inhibition of DNA gyrase and topoisomerase IV [60] and so is most potent when inside bacterial cells. Despite a possible decrease in cell permeability, CDN11 was still able to reduce bacterial numbers in all models tested. Therefore, in chemically linking an antibiotic to nitroxide(s), there may be some loss in activity against planktonic bacteria but an enhancement of activity against biofilms formed on abiotic surfaces, as reported here. We also speculate that CDN11 might have reduced activity against UTI89 biofilm-like cells (IBC and filamentous) present in cell infection assays and murine bladders as a result of its reduced mammalian cell permeability. 

A challenge in designing effective antimicrobials for UTI is catering to the different niches and forms of UPEC during pathogenesis. Drugs that can access intracellular UPEC would be desirable, as extracellular UPEC can be more readily accessed by antibiotics and immune cells and also voided through urine. Additionally, drugs with antibiofilm activity would be beneficial for targeting catheter biofilms and IBCs. CDN11’s reduced activity in cellular infection models lends itself to being better purposed as a topical antibiofilm agent, as it had better activity in the catheter biofilm model. However, CDN11 could be further chemically modified to attempt to address these shortcomings, given its ability to reduce recoverable bacteria in all models. Alternatively, first-line antibiotics such as fosfomycin and nitrofurantoin [25] have yet to be hybridised with nitroxides and present an unexplored avenue for UTI antibiofilm agents. To date, several other antimicrobial agents have been hybridised to nitroxides and have shown similar trends in antibiofilm activity against their target species [19,61,62]. Thus, paired with our data, nitroxide antibiotic hybridisation is a promising strategy for eradicating biofilms, especially in infections that involve biofilms or biofilm-like structures. However, in order to design and synthesise candidates with a high chance of therapeutic success, more fundamental investigations need to be conducted into the mode of action of nitroxides. Understanding how nitroxides impact biofilms will be key to designing better therapeutics that will reflect better efficacy in preclinical models, allowing for faster translation into human trials.

## 4. Conclusions

In conclusion, we present here a robust pipeline for preclinical drug testing, using the nitroxide–ciprofloxacin hybrid CDN11 as a candidate. Testing CDN11 in a pipeline designed to replicate the clinical aspects of urinary tract infection revealed promising activity against uropathogenic bacterial biofilms formed on abiotic surfaces, such as urinary catheters. Antibiofilm activity was also observed in infected bladder cells and tissues, but CDN11 activity was not superior to the parent antibiotic ciprofloxacin, possibly due to its increased drug size. Future work could test CDN11’s potential to eradicate UPEC and other bacterial biofilms on various healthcare surfaces or as a topical treatment or catheter coating, where it would have direct access to the biofilm. New antibiotic development is arduous and requires large investments of time and money [63]. As new therapeutic compounds and antibiotics become available for testing, they should be streamlined through tailored preclinical pipelines, so that the most promising drugs reach clinical trial sooner and new antibiofilm agents become available faster for clinicians.

## 5. Materials and Methods

### 5.1. Ethics Statement

All UTI mouse work was conducted under QIMR-B ethics, approval number A1703-600M, and Queensland University of Technology OGTR, approval number 1700000118. 

### 5.2. Bacterial Strains and Culture Conditions

UPEC strain UTI89 [64] was used in this study as a reference cystitis strain, and EC958 [65] as a multidrug-resistant strain. Unless otherwise stated, all bacteria were routinely maintained and cultured in Luria broth (LB) media at 37 °C, 200 rpm (1% *w*/*v* tryptone (Oxoid, CM0087), and 0.5% *w*/*v* yeast extract (Gibco, 212750), 0.5% *w*/*v* salt) from a single colony on a plate (1.5% *w*/*v* agar (BD, DF0140-01-0)), 37 °C. For all antimicrobial activity assays, testing was conducted in Mueller–Hinton (MH) medium (30% *w*/*v* dehydrated beef infusion, 1.75% *w*/*v* casein hydrolysate, 0.15% *w*/*v* starch, Oxoid, CM0405).

### 5.3. Antimicrobial Compounds

Ciprofloxacin (Sigma, 17850) stocks were routinely made from powder and dissolved in 0.1 M HCl (30 mM, 10 mg/mL). For MIC assays, ciprofloxacin was used at a concentration range of 3000 μM–0.0023 μM. For MBEC assays, ciprofloxacin was used at a concentration range of 800 μM–1.57 μM. For all other assays, ciprofloxacin was used at a concentration of 400 μM.

CDN11 was synthesised as previously described [22]. Briefly, the EDC-promoted amide coupling of ciprofloxacin ethyl ester with Boc-Lys(Cbz)-OH gave the fully protected conjugate, which was deprotected via hydrogenolysis at the α-amine and reacted in another EDC amide coupling reaction with Boc-Lys(Cbz)-OH. The deprotection of the two Cbz-protected amines and subsequent amidation with 4-carboxy-2,2,6,6tetramethylpiperidine 1-oxyl (CTEMPO) gave the nitroxide-functionalised ciprofloxacin, which was deprotected through base-mediated ester hydrolysis, followed by Boc-deprotection, to give CDN11. Stocks were routinely made from powder and dissolved in 100% DMSO (10.5 mM, 10 mg/mL). For MIC assays, CDN11 was used at a concentration range of 800 μM–2.63 μM. For MBEC assays, CDN11 was used at a concentration range of 800 μM–1.57 μM. For all other assays, CDN11 was used at a concentration of 400 μM.

### 5.4. Minimum Inhibitory Concentration (MIC) Assay

MICs for nitroxides, antibiotics, and hybrid compounds were performed as described by the Clinical and Laboratory Standards Institute (CLSI) [66]. Briefly, in a 96-well plate, twofold serial dilutions of each compound were prepared to a final volume of 100 μL in MH medium (CDN11: 800 μM–2.63 μM, CIP: 3000 μM–0.0023 μM), with a matched DMSO vehicle control for the highest concentration. Each well was inoculated with 100 μL bacteria suspension in MH containing 5 × 10^6^ bacterial colony forming units (CFUs), prepared from overnight cultures in LB. The MIC for a compound was defined as the lowest concentration that prevented visible bacterial growth after 18 h of static incubation at 37 °C. MICs were performed a minimum of two times with three technical replicates per condition.

### 5.5. Minimum Biofilm Eradication Concentration (MBEC) Assay

MBEC assays were performed as per the manufacturer’s protocol with a Calgary biofilm device (CBD) (Innovotech Inc., Edmonton, Canada). The CBD consists of a 96-well plate and an associated lid consisting of small protrusions (pegs) that are immersed in each well when the lid is covered, facilitating individual biofilm growth on each peg. Briefly, UTI89 grown for 18 h in LB were diluted to 1 × 10^7^ CFU/mL in LB and used to inoculate the plate with 130 μL of culture per well. The CBD was incubated for 24 h with shaking (150 rpm) at 37 °C in 95% relative humidity. Following 24 h of growth, biofilms were washed once in PBS (130 µL) by incubating statically for 10 s to remove planktonic cells and then the peg lid was transferred to a new 96-well plate containing ciprofloxacin and CDN11 in MH, in concentrations ranging from 800 μM to 1.57 μM, with a matched HCl/DMSO vehicle control for the highest concentration, respectively. The plate was then incubated for a further 24 h at 37 °C with shaking (150 rpm), then washed once in PBS (200 µL, 10 s static incubation), and sonicated in a water bath (Elma Ultrasonic Cleaner S10H) for 20 min at 20 °C to suspend the biomass in 200 µL of PBS. Each well was serially diluted tenfold and 5 μL was spotted onto LB agar plates in triplicate to determine CFU recovered from each peg biofilm. Plates were incubated overnight at 37 °C with colonies counted the following day to obtain log_10_(CFU/mL) values for each treatment condition. Biofilm experiments were performed a minimum of two times, with 3–8 pegs per group. MBEC values are reported as the concentration at which there are no recoverable biofilm CFU (complete eradication).

### 5.6. Catheter Biofilm Model

The in vitro catheter biofilm assays were performed similar to the MBEC assay. UTI89 grown for 18 h in LB were diluted to 1 × 10^7^ CFU/mL in LB and 400 μL/well used to inoculate a 48-well plate. Each well contained catheters made from ~3 mm sections of UV-sterilised polyethylene tubing (BD, 0.28 mm inner diameter, 0.61 mm outer diameter). Plates were incubated for 24 h with shaking (150 rpm) at 37 °C in 95% relative humidity. Following 24 h of growth, catheters were washed once in PBS (400 μL) to remove planktonic bacterial cells and then were transferred to a new plate containing CTEMPO, ciprofloxacin, and CDN11 in MH, at a concentration of 400 μM each, with matched vehicle controls. Catheters were then incubated for a further 24 h at 37 °C with shaking (150 rpm), and then washed once in PBS (400 µL), sonicated, and processed as above to determine CFU. Catheter experiments were performed a minimum of two times, with 4 catheters per group.

### 5.7. Human Cell Infection Assays

Type 1 fimbriae (T1F)-enriched UTI89 was routinely prepared and cultured from highly fimbriated stocks, which were incubated overnight statically at 37 °C for 18 h. Bladder epithelial cells T24 (ATCC HTB-4), were maintained in Roswell Park Memorial Institute (RPMI) 1640 medium (Invitrogen), supplemented with 5% heat-inactivated foetal bovine serum (Invitrogen) at 37 °C. Adhesion and invasion assays with UPEC strain UTI89 were performed as previously described [30]. Briefly, T24 cells were seeded at a density of 5 × 10^5^ in 24-well plates and then grown overnight to form confluent monolayers. Confluent T24 monolayers were infected with UTI89 for 1 h at 37 °C at a multiplicity of infection (MOI) of 10 (5 × 10^6^ CFU/well), then washed with PBS. For adhesion assays, monolayers were then incubated with either RPMI only (untreated), DMSO/0.1 M HCl (vehicle controls), 400 μM ciprofloxacin (antibiotic), or 400 μM CDN11 (dinitroxide–ciprofloxacin antibiotic hybrid) for 2 h at 37 °C. For invasion assays, infected monolayers as above were first incubated with 200 μg/mL gentamicin for 1 h to eliminate extracellular bacteria, then washed three times with PBS. Monolayers were then treated with the same compound groups as the adhesion assays. Post treatment, both monolayers were washed three times with PBS followed by lysis with 500 μL 0.1% Triton X-100 in PBS for 10 min at 37 °C. CFU recovered from each monolayer were determined through serial dilution plating onto LB agar plates. All compound working solutions were made up in RPMI to a concentration of 400 μM (40× MIC of CDN11). Human cell infection experiments were performed a minimum of two times, with 5–6 monolayers per group.

### 5.8. In Vivo UTI Mouse Model and Ex Vivo Bladder Antibiotic Treatment

The 6–7-week-old female C3H/HeJ mice were catheterised as previously described [53]. Briefly, mice were anaesthetised using isoflurane inhalation and catheterised with ~1–2 × 10^8^ CFU in 30 μL of UTI89 in PBS. T1F-enriched UTI89 was prepared as above. The prepared inoculum was deposited directly into the bladder using a sterile polyethylene catheter (same material as in vitro testing) followed by immediate removal [53]. C3H/HeJ mice were sourced from the ARC (Animal Resource Centre, Murdoch WA, Australia). Mice were sacrificed at 16 h post inoculation and the bladders were extracted. 

Bladders were bisected to expose the lumen and were washed in PBS for 5 min in a well of a 96-well plate (one mouse per well). Bladder halves were then transferred to fresh wells in another plate containing 200 μg/mL gentamicin in PBS and incubated for 1 h 37 °C to remove extracellular bacteria, then washed again in PBS. Each bladder half was then incubated with either PBS only (untreated), DMSO (vehicle control), 400 μM ciprofloxacin (antibiotic), or 400 μM CDN11 (nitroxide-functionalised antibiotic) for 6 h at 37 °C. Bladders were treated for 6 h with CDN11 to simulate the half-life of ciprofloxacin in the human body, while the dosage (400 μM) roughly equated to half the therapeutic ciprofloxacin dose commonly prescribed to adults for acute UTIs, while also being the MBEC value determined in vitro [54,55]. Following treatment, bladders were immediately transferred to tubes containing metal beads for homogenisation. Bladders were homogenised in 50 μL of PBS using a Mini Bead beater (BioSpec Products, Bartlesville OK, United States) before topping up to 1 mL with PBS and aliquoting 200 μL of each sample into a 96-well plate. Samples were serially diluted and 5 µL of each well for each sample was plated onto LB agar in quadruplicate and then incubated overnight at 37 °C. The following day, colonies were enumerated, and bacterial CFU was expressed as the log_10_(CFU/0.1 g) of tissue. The ex vivo experiment was performed once, with 8 bladders per group.

### 5.9. Statistical Analysis

All statistical analysis was performed using GraphPad Prism Version 9 software (GraphPad Software). An ordinary one-way ANOVA with Dunn’s multiple comparisons correction was used to test for statistically significant differences between CDN11-treated groups and control group means. Statistical significance was set at *p* < 0.05.

## Data Availability

The authors confirm that the data supporting the findings of this study are available within the article.

## Supplementary Materials

The following supporting information can be downloaded at: https://www.mdpi.com/article/10.3390/antibiotics12101479/s1, Figure S1: Summary of minimum inhibitory concentration (MIC) assays for CDN11 and ciprofloxacin, against UPEC strains UTI89 and EC958.
