# Supplementary material for: Preclinical Evaluation of Nitroxide-Functionalised Ciprofloxacin as a Novel Antibiofilm Drug Hybrid for Urinary Tract Infections
Source: Antibiotics (Basel). 2023 Sep 22;12(10):1479. doi: 10.3390/antibiotics12101479 (PMC10604439; doi:10.3390/antibiotics12101479)
Supplement: Supplementary file 1 [file antibiotics-12-01479-s001.zip › antibiotics-2609680-supplementary.pdf]

Supplementary Material

EC958			UTI89			EC958			UTI89		
400	400	400	84	84	84	128	128	128	0.24	0.24	0.24
200	200	200	42	42	42	64	64	64	0.12	0.12	0.12
100	100	100	21	21	21	32	32	32	0.06	0.06	0.06
50	50	50	10.5	10.5	10.5	16	16	16	0.03	0.03	0.03
25	25	25	5.25	5.25	5.25	8	8	8	0.015	0.015	0.015
12.5	12.5	12.5	2.63	2.63	2.63	4	4	4	0.0075	0.0075	0.0075
G	G	G	G	G	G	G	G	G	G	G	G
D	D	D	D	D	D	HCl	HCl	HCl	HCl	HCl	MH
CDN11						CIP					

**Legend** (concentrations in µM)
[ ] - 0% growth, [ ] - 20% growth, [ ] - 50% growth, [ ] - 100% growth
G – growth, bacteria in media only
D/HCl – vehicle control, **D**MSO or 0.1M **HCl**
MH – media only (Mueller Hinton)

Supplementary Figure 1. Summary of minimum inhibitory concentration (MIC) assays for CDN11 and ciprofloxacin, against UPEC strains UTI89 and EC958. MIC values were determined as per the CLSI standards [1]. The MIC for a compound was defined as the lowest concentration that prevented visible bacterial growth after 18 h of static incubation at 37°C. MICs were performed a minimum of two times with three technical replicates per condition.

References

1. Clinical and Laboratory Standards Institute (CLSI). *Performance Standards for Antimicrobial Susceptibility Testing*, 28th ed.; Clinical and Laboratory Standards Institute, 950 West Valley Road, Suite 2500, Wayne, Pennsylvania 19087 USA. , 2018.
